# Selective chronic recording in small nerve fascicles of sciatic nerve with carbon nanotube yarns in rats

**DOI:** 10.1088/1741-2552/ad1611

**Published:** 2024-01-04

**Authors:** B P Kotamraju, Thomas E Eggers, Grant A McCallum, Dominique M Durand

**Affiliations:** 1 Case Western Reserve University, Neural Engineering Center, Biomedical Engineering, Cleveland, OH, United States of America; 2 Department of Neurosurgery, Emory University, Atlanta, GA, United States of America

**Keywords:** peripheral nerve interfaces, ENG, signal to noise ratio, mutual information, Shannon entropy, carbon nanotube yarns

## Abstract

*Objective*. The primary challenge faced in the field of neural rehabilitation engineering is the limited advancement in nerve interface technology, which currently fails to match the mechanical properties of small-diameter nerve fascicles. Novel developments are necessary to enable long-term, chronic recording from a multitude of small fascicles, allowing for the recovery of motor intent and sensory signals. *Approach*. In this study, we analyze the chronic recording capabilities of carbon nanotube yarn electrodes in the peripheral somatic nervous system. The electrodes were surgically implanted in the sciatic nerve’s three individual fascicles in rats, enabling the recording of neural activity during gait. Signal-to-noise ratio (SNR) and information theory were employed to analyze the data, demonstrating the superior recording capabilities of the electrodes. Flat interface nerve electrode and thin-film longitudinal intrafascicular electrode electrodes were used as a references to assess the results from SNR and information theory analysis. *Main results*. The electrodes exhibited the ability to record chronic signals with SNRs reaching as high as 15 dB, providing 12 bits of information for the sciatic nerve, a significant improvement over previous methods. Furthermore, the study revealed that the SNR and information content of the neural signals remained consistent over a period of 12 weeks across three different fascicles, indicating the stability of the interface. The signals recorded from these electrodes were also analyzed for selectivity using information theory metrics, which showed an information sharing of approximately 1.4 bits across the fascicles. *Significance*. The ability to safely and reliably record from multiple fascicles of different nerves simultaneously over extended periods of time holds substantial implications for the field of neural and rehabilitation engineering. This advancement addresses the limitation of current nerve interface technologies and opens up new possibilities for enhancing neural rehabilitation and control.

## 1. Introduction

Amputation is recommended for individuals with traumatic injuries and circulatory disorders [1]. As of 2017, approximately 57.7 million people worldwide were living with traumatic amputations, and an estimated 75 850 prosthetics are required annually to cater to amputees [2]. Despite the advanced prosthetic devices available, individuals with traumatic amputations face challenges in fully restoring limb functionality, which negatively impacts their quality of life. The main hurdle in prosthetic device development lies in the absence of a comprehensive control signal capable of providing the necessary degrees of freedom. Although these neural signals exist within the nerves, their recording proves difficult.

Peripheral nerve interfaces (PNI’s) have been utilized since the 1960s for various purposes, including diaphragmatic pacing, urinary incontinence, chronic pain alleviation, and muscle activation in paralyzed individuals and those with spinal cord injuries [3–6]. However, despite technological advancements, the ability to selectively record activity in small fascicles of peripheral nerves over extended periods remains a challenge. One commonly used PNI is the cuff electrode, which records local field potentials outside the epineurium [7–11]. Although these electrodes are relatively safe due to their placement outside the nerve, they have lower signal-to-noise ratios (SNR) compared to electrodes that penetrate the epineurium and/or perineurium. Intrafascicular electrodes, such as the longitudinal intrafascicular electrode (LIFE), Utah slanted electrode array (USEA), and transverse intrafascicular multichannel electrode (TIME), have been extensively studied to overcome the limitations of extraneural electrodes like cuff electrodes [12–19]. Intrafascicular electrodes offer improved SNR by being closer to the signal source, as well as enhanced subfascicular selectivity and signal discrimination. However, they can cause damage to nerve fibers and intraneural blood vessels, resulting in poor long-term performance [3].

Previous research has demonstrated that flexible electrodes like carbon nanotube yarn (CNTY), which possess a flexural rigidity similar to small nerves, can produce reliable and stable chronic recordings in the autonomic nervous system [20–22]. Based on this, we hypothesize that CNTY electrodes can also offer high SNR for recording neural activity in the peripheral somatic nervous system over extended periods. The objective of this study is to investigate the use of CNTY electrodes in the peripheral somatic nervous system and assess the quality and stability of the recorded signals. We employ information theory metrics, such as Shannon entropy, to measure the information content of the signals obtained with the CNTY electrode. While information theory is commonly used in neuroscience to quantify information content, its application in evaluating the information content of peripheral neural signals is not as prevalent in current literature. We evaluate the information content obtained from CNTY electrodes in relation to one of the few studies that analyzed the information content of peripheral nerve recordings using the flat interface nerve electrode (FINE) electrode. Impedance values and signal selectivity of CNTY electrodes for stimulation have been reported in previous studies [21]. This study will assess signal selectivity for recording chronic neural signals by determining the amount of shared information between the recorded signals of individual fascicles, measured through mutual information.

## 2. Methods

### 2.1. CNTY electrode

**Figure 1.**
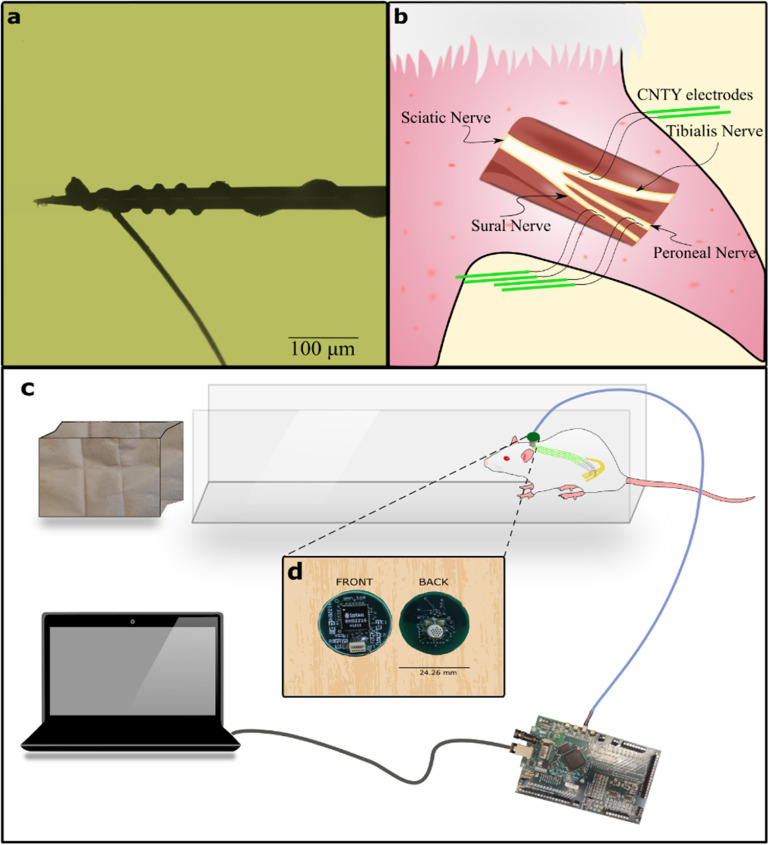
Illustration of electrode design, implantation process, and experimental setup. (a) Depicts the use of insulated CNT yarn wound around a tungsten microneedle carrier to facilitate the electrode’s insertion into individual fascicles. (b) The sciatic nerve is subdivided into three distinct fascicles: peroneal, tibial, and sural. Each of these fascicles is implanted with a pair of electrodes, as shown in (a), placed approximately 1 cm apart, resulting in a total of six electrodes within the nerve. (c) Shows the experimental setup, with the animal positioned at the end of a walkway to encourage walking toward a dark enclosure. (d) The recording board containing an INTAN chipset is connected to a percutaneous port on the rat’s back and linked to an FPGA via a USB cable on the opposite end.

The CNTY electrodes were made using two different material types, CNT Yarns made from high rate spun vertically aligned multiwalled CNT (VA-MWCNT) arrays [23] and a stainless steel 35NLT-DFT wire (Fort Wayne Metals). The two were joined using a conductive epoxy resin (H20E, EPO-TEK), and this junction was placed within a Dacron mesh for structural stability and insulated using a silicone elastomer (MED- 4211/MED-4011, NuSil Silicone Technology). The entire CNTY electrode was coated with a 5 *μ*m Parylene-C coating (vapor deposition coating, SMART Microsystems). To obtain a recording site, ∼200–500 *μ*m in length with a surface area ∼(1.5–3.9)*10^−9^ m^2^ of the insulation was removed from the CNT end which was wound around the tip of a tungsten microneedle (FHC inc, part no# UEWSGKSNXNND) (figure 1(a)) as described previously [21].

### 2.2. Surgical procedure

The Case Western Reserve University Institutional Animal Care and Use Committee approved the surgical and experimental protocol for this study, ensuring compliance with animal welfare laws and regulations (protocol Number: 2016-0328). Six male Sprague-Dawley rats, aged between 7–12 weeks, were selected for electrode implantation.

To expose the rat sciatic nerve, a longitudinal incision was made along the femoral axis, and the superficial muscles covering the nerve were separated (figure 1(b)). The sciatic nerve was then exposed for approximately 2–3 mm proximal to the point where it branches into individual fascicles by dissecting the epineurium. Using a micro hook (FST Item no 10 062-12), the individual fascicles were gently held with tension, and a tungsten microneedle with the electrode was inserted through the perineurium to position the electrode contact within the fascicle. This process was repeated to place two electrodes within each of the three fascicles of the sciatic nerve for differential recording. Following implantation, a silicone cuff electrode was placed around the implant site. This cuff did not contain any recording electrodes but had an exposed metal (gold) surface to reduce electromyogram (EMG) interference [24–26]. After closing the cuff, approximately 1 ml of fibrin glue (Tisseel, Baxter International Inc.) was applied to the implantation site to secure the electrodes in place. The DFT ends of the electrodes were routed to the rat’s back, where they were soldered to a connector (Omnetics connector corporation MCP-5-SS (#A22000-001)). The connector was insulated and attached to a circular Dacron mesh with a radius of approximately 2 cm using UV curing epoxy (EPO-TEK OG603). The Dacron mesh was then sutured to the back muscles, ensuring the port was securely positioned on the back of the specimen. The amplifier ground was placed underneath the skin on the dorsal side of the animal, where neural activity is absent. Electrodes were successfully implanted in six rats for varying durations, ranging from 1 week to 12 weeks. The number of electrodes that were successfully implanted varied among the rats. In certain cases, rats received electrodes in two out of three fascicles, primarily as a result of electrode-related complications during the implantation process. These complications were further categorized into two types: surgical failure, which involved the disengagement of the CNTY electrode from the needle with no possibility of reengagement, and mechanical failure, which entailed the snapping of the CNTY electrode during implantation.

### 2.3. Experimental setup

Implanted rats were allowed to recover for a week after surgery before neural recordings were performed. The rat was placed in an elongated 3 feet walkway that is 10 cm wide (figure 1(c)). Where the rat was allowed to move from one end to the other. For each experiment, the rat walked spontaneously along the runway into a dark enclosure and was then placed back at the beginning of the runway. Randomization, blinding or replication techniques were not employed as they were not relevant to the study.

### 2.4. Recording

A recording system consisting of differential amplifiers, multiplexers, A/D converters (INTAN RHD2216 chipset), and Omnetics connector (MCS-5-SS (#A22001-001)) was built (figure 1(d)). This board connected to the port on the back of the rat enabling us to record differentially from the electrodes. The recording board employed four-channel hardware averaging technique to increase the SNR [27]. The recording board connects to a FPGA board made by Opal Kelly (XEM6010-LX45) which runs the acquisition hardware for the INTAN recording system. The neural signals were band pass filtered between 100–7500 Hz and sampled at 20 kHz.

### 2.5. Signal processing

**Figure 2.**
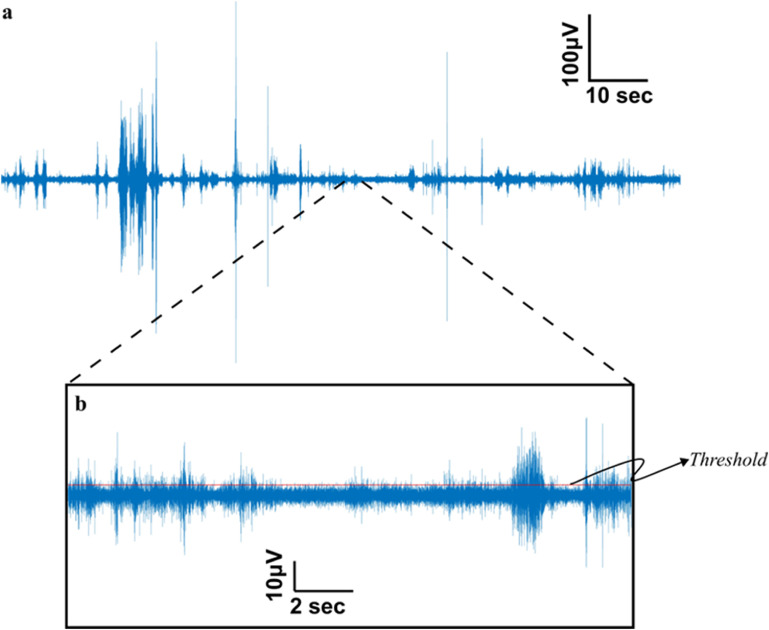
Representative neural signal obtained from the individual fascicle of the sciatic nerve (a). Neural signal obtained from the tibialis fascicle of a rat sciatic nerve during locomotion is subjected to bandpass filtering between 500–1200 Hz enhancing the SNR of the recorded signals. (b). The SNR calculation (equation (1)) involves the determination of the threshold for identifying positive spike amplitudes within the signal. This threshold is established as three times the root mean square (RMS) value of the baseline. The baseline, defined as the quietest segment of the signal, typically representing a region with minimal or no discernible neural activity, as illustrated in the central segment above the scale.

The electroneurogram (ENG) data from the INTAN RHD format was imported into MATLAB^®^ (v2021a Mathworks^®^) for post signal processing. It is important to note that a prior study [21] established that CNTY electrodes, when in motion, do not introduce any motion artifacts into neural recordings. Additionally, the recordings were digitized as close to the source as possible, specifically at the percutaneous port on the back of the rat, in order to minimize other sources of motion artifact during the recording process. These signals were subsequently band-pass filtered between 500–1200 Hz to minimize noise from EMG and other sources such as white noise, triboelectric noise, and electromagnetic noise. These filtered signals (figure 2(a)) were then used to calculate the SNR, entropy, and mutual information measures discussed in the later sections. The SNR of the signal was calculated using the formula shown in equation (1),

\begin{align*}&amp;SNR \nonumber\\ &amp;= 20{{\text{log}}_{10}}\frac{{\frac{1}{N}\mathop \sum \nolimits _{i = 1}^Namplitudes{\text{ }}of{\text{ }}peaks{\text{ }}above{\text{ }}threshold}}{{base{\text{ }}line{\text{ }}RMS}}\end{align*}

The quietest period (the period within the signal that has the least amount of activity as shown by the middle section of figure 2(b)) of the recorded signal was chosen as the baseline duration, where the rms of the signal was less than 12 *μ*V. The threshold of the signals was determined as 3 times the baseline RMS value. All the spikes above the threshold were considered neural spikes for SNR and Information theory metric calculations. For the SNR calculations the positive amplitude of the spikes were used. Using the information theory toolbox developed by Timme and Lapish [28], Information theory metrics like Shannon entropy and mutual information were computed, with spike counts serving as the encoding variable. To perform these calculations, the spike counts were partitioned into 32 uniform width bins i.e. 32 states. The Shannon entropy (H(X)) is determined as illustrated in equation (2), with ‘*x*’ representing the spike count state and ‘*p*(*x*)’ denoting the likelihood of a specific state occurring,

\begin{align*}H\left( X \right) = - \sum\limits_{x \in X} p\left( x \right)\log \left( {p\left( x \right)} \right).\end{align*}

In a similar fashion, mutual information (*I*(*X;Y*)) is determined following the formula in equation (3), where ‘*X*’ denotes the spike count state in one fascicle, and ‘*Y*’ indicates the spike count state in the other fascicle. Additionally, ‘*P*(*x, y*)’ represents the joint probability of both ‘*X*’ and ‘*Y*’ states occurring,

\begin{align*}I\left( {X;Y} \right)&amp; = H\left( X \right) - H\left( {X{\text{|}}Y} \right) \nonumber\\&amp;= - \sum\limits_{x \in X,y \in Y} p\left( {x,y} \right)\log \left( {\frac{{p\left( x \right)p\left( y \right)}}{{p\left( {x,y} \right)}}} \right).\end{align*}

### 2.6. Statistical testing

To assess the mean SNRs of the three distinct fascicles, a multi-comparison test was conducted using the Tukey–Kramer procedure with a significance value set at 0.05. For the regression analysis of SNR and entropy, the trendline slope was statistically examined at a significance value of 0.05 to determine whether it significantly deviated from zero. To evaluate whether the mutual information between pairs of fascicles differed significantly from that of independent fascicles, one-sample two-sided t-tests were performed at a significance value of 0.05 to determine if the mean mutual information between these pairs was equal to zero or not.

## 3. Results

### 3.1. Quantifying signal quality and information content

**Figure 3.**
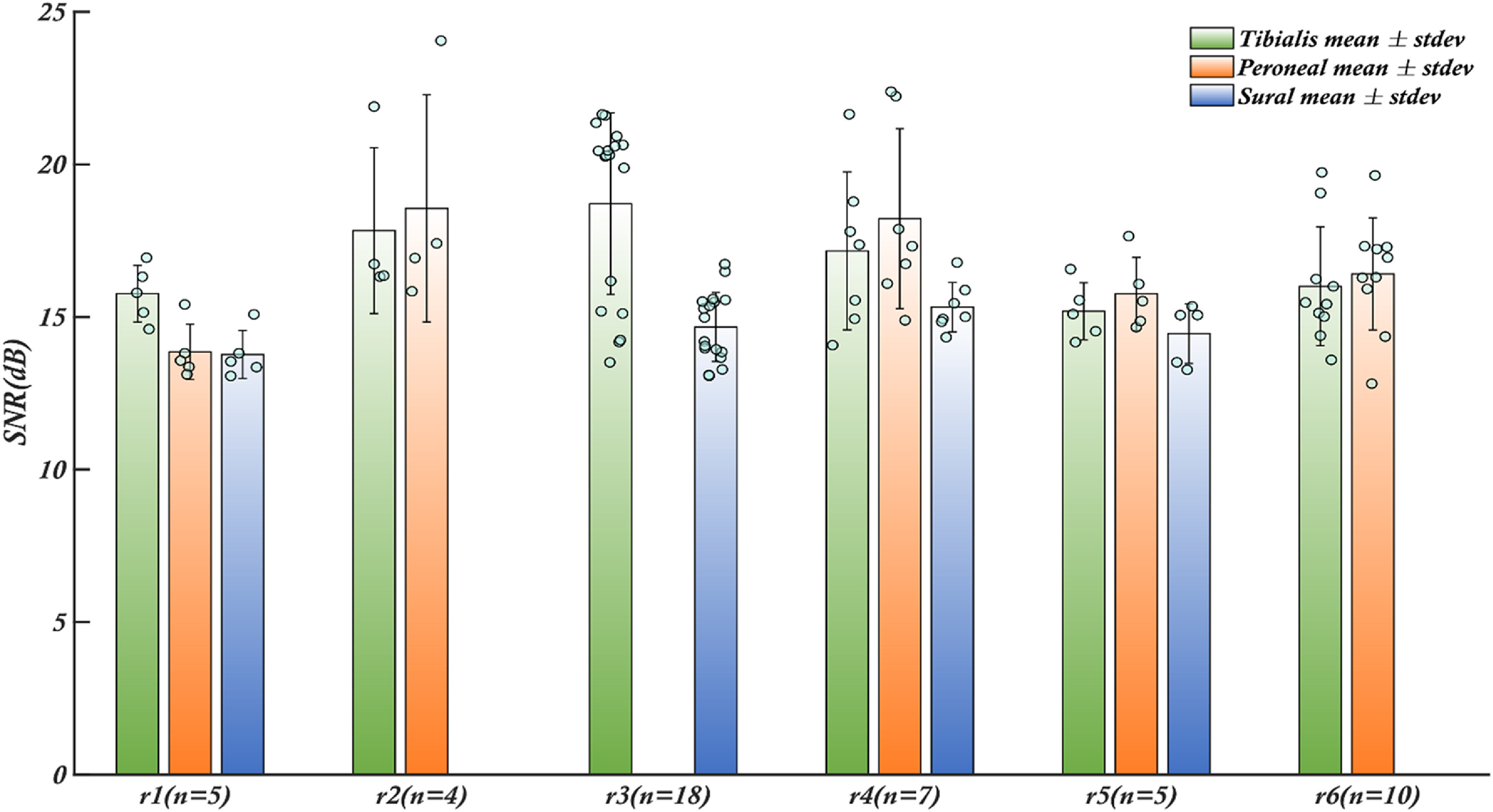
Robust SNR performance of CNTY electrodes in rats. Throughout the duration of the recording sessions, the CNTY electrodes consistently exhibited a noteworthy SNR for each rat, with values surpassing the 6 dB threshold. The *x*-axis represents distinct fascicle clusters for individual rats, denoted as R1 to R6, while ‘n’ quantifies the number of recordings carried out for each rat during the implantation period. Remarkably, the performance of each electrode, situated within the individual fascicles, consistently yielded signals with an SNR exceeding 13 dB across all subjects. The bars represent the mean values of SNR for each fascicle, the error bars are the respective standard deviations. Individual data points contributing to the mean are shown as blue dots.

**Figure 4.**
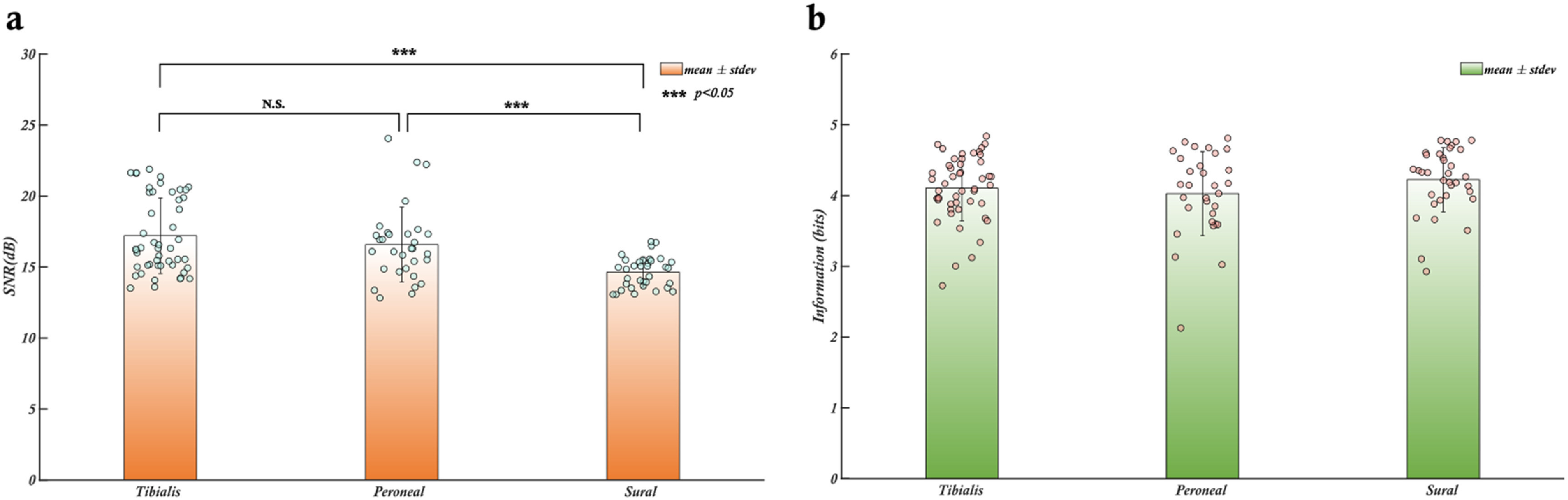
SNR and information content of neural signals from individual fascicles of the rat sciatic nerve (a). The SNR of the Tibialis and Peroneal fascicles are significantly higher than the SNR of the Sural fascicle, however, all three fascicles have a mean SNR greater than 13 dB demonstrating that the electrode performance is reliable and stable across different fascicles. (b). The mean amount of information obtained from each individual fascicle of the rat sciatic nerve is approx. Four bits for a total of 12 bits of information from the whole nerve. The bars represent the mean values of SNR/entropy for each fascicle, the error bars are the respective standard deviations. Individual data points contributing to the means are shown as dots.

The signal to noise ratio (SNR) is a commonly used measure to assess the quality of recorded biomedical signals obtained from a recording electrode. A high SNR signal is desirable for sensory and motor restoration applications as it facilitates signal identification and extraction of information. CNTY electrodes have demonstrated high SNR recordings of individual fascicles, as illustrated in figure 2, which displays a bandpass filtered neural recording from the tibial fascicle of the sciatic nerve with an SNR value calculated using equation (1). Positive spike amplitudes were determined using a thresholding algorithm. To evaluate SNR throughout the implantation period, the mean and standard deviation of SNR were determined for each individual rat, as depicted in figure 3. This analysis was conducted for each animal (*N* = 6). In all the rats examined, the SNR for the tibialis fascicle measured 17.21 ± 2.64 dB. Similarly, the peroneal fascicle exhibited an SNR of 16.58 ± 2.59 dB, while the sural fascicle showed an SNR of 14.64 ± 1.06 dB (figure 4(a)).

In the case of thin-film longitudinal intrafascicular electrode (tf-LIFE) electrodes, which have been widely used and even implanted in human median and ulnar nerves, the maximum SNR before applying a denoising algorithm is 6 dB(calculated as 20*log_10_(max(reported SNR values))) [13]. Although it is not feasible to make a direct comparison between the signal quality of CNTY electrodes and tf-LIFE electrodes due to the differing experimental setups in the two studies, our study focuses on SNR measurements obtained from the individual fascicles in the sciatic nerve of rats with a bandpass frequency of 500–1200 Hz. In contrast, the previously cited study involving tf-LIFE electrodes captured neural recordings in human median and ulnar nerves with a bandpass frequency range of 100–10 kHz. Nevertheless, the aforementioned tf-LIFE study can serve as a valuable reference point for drawing reasonable inferences regarding the recording capabilities of the CNTY electrode. Across all six rats, all the electrodes consistently exhibited a mean SNR greater than 13 dB throughout the recording period, with the maximum standard deviation of 3.2 dB observed in one rat. The SNR values observed for each fascicle in all rats exceeded the maximum SNR of 6 dB reported for tf-LIFE electrodes, hinting at the potential for CNTY electrode recordings to match or even surpass the quality of those from tf-LIFE electrodes. Additionally, no significant difference in SNR was observed between the recorded signals from the tibial and peroneal fascicles when comparing the SNR values across all rats (figure 4(a)) with *p*-values > 0.05.

While the SNR provides a valuable measure of signal quality, it does not indicate the amount of information that can be inferred from the signal. To address this, we employed Shannon Entropy, a widely used information theory measure, to determine the information content of the signal or dataset [28]. For each fascicle in each rat trial, we calculated the Shannon Entropy and determined the mean and standard deviation of the information content across all rats and fascicles. To facilitate comparison, entropy values were computed with a base value of 2, resulting in units of bits. The maximum information content of 3 bits was reported for the FINE electrode [25, 29], a well-studied electrode in the literature [3] that has also been used to calculate information transfer rate from peripheral neural recordings in the sciatic nerve of dogs. The 3 bits of maximum information was calculated from the maximum number of states used for the information transfer rate calculations. Since the maximum number of states used was 8, the maximum information would be log_2_(8) = 3 bits. The entropy analysis indicated an average of 4 bits per fascicle, totaling 12 bits for the entire nerve using the CNTY electrode. While direct comparisons are not possible due to differences in animal models and experimental setups, the results suggest that CNTY electrodes provide neural recordings with more information than FINE electrodes [25, 29] (figure 4(b)).

### 3.2. Signal stability over time

**Figure 5.**
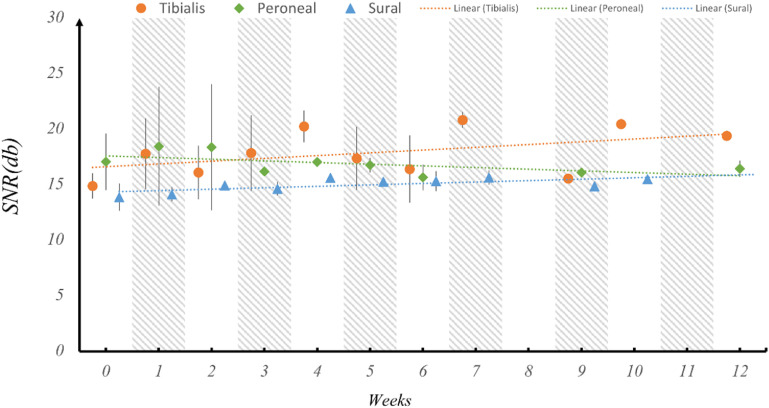
Stability of mean SNR over 12 weeks post-implantation across fascicles. Mean SNR values for each fascicle were computed on a weekly basis over a maximum period of 12 weeks. Subsequently, a regression analysis was conducted to assess the significance of the trend line’s slope, aiming to detect any noteworthy changes in electrode performance. The SNR remained consistent over the 12-week post-implantation period, with minimal fluctuations, maintaining an average level of approximately 15–18 dB.

**Figure 6.**
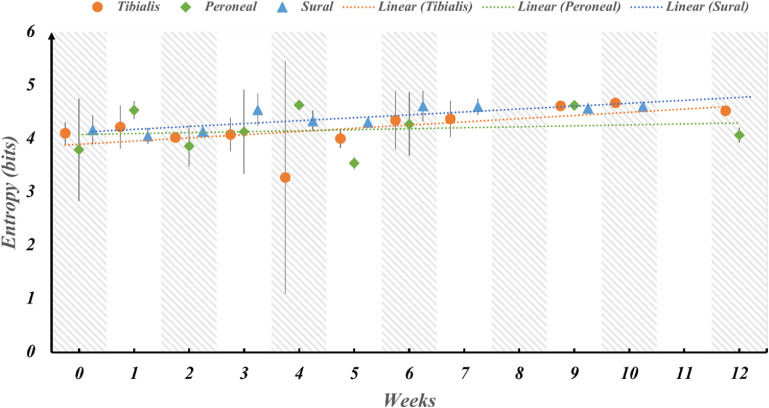
Stability of information content over 12 weeks post-implantation across fascicles. The entropy of the signal, representing the mean information, was computed for each fascicle in a series of weekly intervals extending up to 12 weeks after implantation in rats. Subsequently, a regression analysis was conducted to assess whether the slope of the trend line significantly deviates from zero. This analysis aims to discern if there is any substantial decline in the informational quality of the signal over time. The results indicate that the information content remains consistently stable, hovering around 4 bits per fascicle, without any noticeable reduction during the entire duration of implantation.

One of the main barriers to the widespread adoption of neural interfaces is the inability of the neural electrodes to provide stable recordings over long-term durations. Neural interfaces suffer from degrading electrode performance due to inflammatory response and encapsulation of the recording site over long periods of time. Therefore, we tested the stability of the SNR of the neural signals during the period of 12 weeks. To assess the electrode performance over time, the SNR of the recordings for each fascicle of the sciatic nerve was averaged across all rats for each week post implantation (figure 5). The SNR remains stable over 12 weeks with minor fluctuations between the weeks for all the fascicles. Similarly, the Entropy of the signal was investigated as a function of time post implantation to determine whether the amount of information that can be inferred from the signals depreciates over time (figure 6). The amount of information (entropy of the signal) also remains stable over 12 weeks with minor fluctuations between the weeks for all the fascicles.

Regression testing was performed on the trendline slope of each fascicle for both the SNR and Entropy to determine whether the slope is significantly less than zero implying a significant change in SNR or Entropy over time. The results of the analysis are tabulated in tables 1 and 2 for SNR and Entropy respectively. The *p*-values indicate that the slopes of the trendlines are not significantly different from 0 which implies that the SNR and Entropy metrics of the signal quality are stable over time.

**Table 1.** Regression testing for the trendline slope of SNR throughout 12 weeks post implantation shows no significant decrease in slope for the implantation duration. The slope for the trend line of sural fascicle is shown as significantly increasing due to the low standard deviation for the sural fascicle, however, the SNR is not decreasing over time indicating no electrode performance degradation.

Fascicle	*P*-value	Slope	Inference
Tibialis	0.15	0.25	*p-value > α_0.05_ so we fail to reject the null hypothesis*
Peroneal	0.09	−0.14	*p-value > α_0.05_ so we fail to reject the null hypothesis*
Sural	0.02	0.12	*p-value < α_0.05_ so we reject the null hypothesis, however the slope is positive so the SNR is not decreasing*

**Table 2.** Regression testing for the trendline slope of entropy throughout 12 weeks post implantation shows no significant decrease in slope for the implantation duration. The slope of the trend line for sural fascicle is shown as significantly increasing due to the low standard deviation for the sural fascicle, however, the entropy is stable over time indicating little or no electrode performance degradation or loss of information from the recorded signals.

Fascicle	*P*-value	Slope	Inference
Tibialis	0.54	0.05	*p-value > α_0.05_ so we fail to reject the null hypothesis*
Peroneal	0.64	0.018	*p-value > α_0.05_ so we fail to reject the null hypothesis*
Sural	0.03	0.05	*p-value < α_0.05_ so we reject the null hypothesis, however the slope is positive so the entropy is not decreasing*

### 3.3. Mutual information between individual fascicles of a nerve

**Figure 7.**
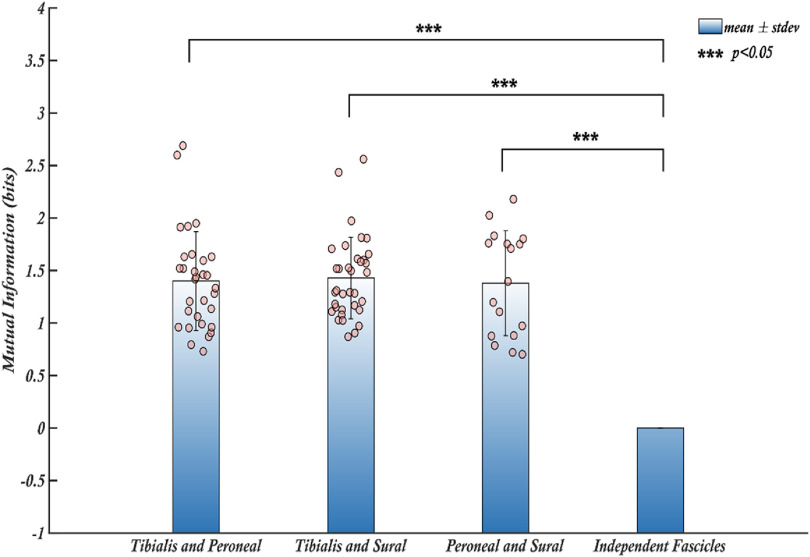
The mean mutual information shared between the pairs of fascicles for all trials. The average mutual information observed among pairs of fascicles across all trials demonstrates that each fascicle shares a substantial amount of information, significantly exceeding zero bits. This finding suggests the presence of shared information among the fascicles and indicates that they are not entirely independent from one another. The bars represent the mean values of mutual information for each fascicle, the error bars are the respective standard deviations. Individual data points contributing to the means are shown as dots.

The selectivity of a recording system measures the capability of each electrode to record unique signals from various sources. A highly selective nerve interface is necessary for a wide range of applications such as motor and sensory restoration. Although fascicular selectivity can be ensured since electrodes are placed surgically in individual fascicles, the information could be shared between fascicles. Therefore, we then calculated the mutual information between the recorded signals and used that metric as an estimate of selectivity. The mutual information between two signals determines the amount of information that can be inferred about a signal from the other i.e. the amount of dependent information that is common between the two. The mutual information between pairs of fascicles for each trial has been calculated using the information theory toolbox developed by Timme and Lapish [28]. The mean mutual information for each pair of fascicles for all rats was then calculated and tested to determine whether the information recorded by the electrodes was independent between the fascicles. The results shown in figure 7 indicate that a mean mutual information of ∼1.4 bits was found between pairs of fascicles.

## 4. Discussion

The ability to record neural signals selectively over an extended period has been difficult due to the degradation of electrode performance and encapsulation at the implantation site, hindering the widespread use of this technology in human patients for sensory and motor restoration. Previous research has shown that CNTY electrodes can be used for long-term interfacing with the rat vagus nerve to record vagal tone and decode vagus nerve activity for various behaviors [20, 22]. The size of individual fascicles in the rat sciatic nerve ranges from 15–300 *μ*m in diameter [30], similar to the size of the rat vagus nerve, which ranges from ∼250–500 *μ*m in diameter [20]. This makes CNTY electrodes suitable for chronic recordings in the somatic nervous system. In the sciatic nerve, CNTY electrodes recorded signals with an SNR 3 times higher than the reported SNR for tf-LIFE electrodes in each channel. Although recording conditions and targeted nerves differed between CNTY and tf-LIFE, the results suggest that CNTY has the potential to improve signal quality recorded from nerves.

Another way to evaluate signal quality improvement is by measuring the amount of information that can be inferred/encoded from the signal using information theory metrics. On average, about 4 bits of information could be retrieved from each fascicle, totaling 12 bits for the sciatic nerve. The FINE electrode was chosen as a reference point for this metric because it is widely used and extensively studied [9, 26, 31–36]. While information theory metrics have been extensively used in neuroscience and neural engineering, limited information is available on information metrics in peripheral nerves. The only other published value of nerve signal entropy was obtained with a 16-contact nerve cuff electrode (FINE), which yielded 3 bits of information from the whole nerve after post-processing [25, 29]. While a direct comparison between the two cannot be made since the experimental design and recording condition are different in the two studies, these results indicate that using CNTY in individual fascicles improves upon extraneural cuff electrodes. A single extra bit allows encoding for a total of 2^4^ = 16 states from a single fascicle instead of 2^3^ = 8 states possible with the FINE electrode for the whole sciatic nerve.

Furthermore, the results offer a compelling observation concerning the pronounced disparities in SNR values between the sural fascicle and the remaining two fascicles of the sciatic nerve. This significant variance can be attributed to the relatively lower number of axons within the sural fascicle in comparison to the tibialis and peroneal fascicles [37]. The increased axon count leads to a greater number of signal sources, consequently resulting in heightened SNR values.

One challenge for long-term chronic neural interfacing is the longevity of electrodes due to foreign body response and fibrous encapsulation, leading to signal degradation. The results show that electrode recording performance remained stable for up to 12 weeks. The mean SNR of neural signals for each fascicle remained stable over time, with trendline slopes not significantly different from zero, indicating no decrease in SNR due to electrode degradation and encapsulation, consistent with previous study results [21]. Currently, there is limited information on recording SNR stability for intrafascicular electrodes such as TIME and tf-LIFE over extended periods. One study reporting chronic recording performance used floating microelectrode arrays (FMAs) with Pt-Ir contacts and showed a significant decrease in SNR and functional contacts over time [38]. Similarly, the amount of information inferred from the signal remained constant over time, indicating no decline in electrode performance or benefits. The results of regression testing on the sural fascicle indicate a positive trendline slope for SNR and entropy. This implies a slight increase in their values over time. However, it is important to note that the significance of this increase is affected by the relatively low standard deviation in SNR and entropy values for the sural nerve, which can distort the results of the regression analysis.

It should be noted that the SNR and entropy analysis over time only involved four out of the six rats in the study. The remaining two rats had only a single recording session and were thus excluded from the analysis of results over time. Additionally, not all rats contributed to the mean SNR and entropy values for each week in the regression analysis because the recordings were not conducted on a weekly basis after the implantation. This irregular recording schedule has the potential to influence some trendlines to appear positive. Previous histological work has indicated that, after 138 d, there was minimal to no significant encapsulation and immune response to CNTY electrodes in the peripheral nervous system [21].

Although acquiring 4 bits of information from each fascicle represents a significant improvement, it may not be useful if the information conveyed is identical across all fascicles. Mutual information is an information theory metric used to measure the amount of information that can be inferred about one signal from another. While heavily used in neuroscience [28, 39, 40], it has been used for the first time to measure shared information between individual fascicles of somatic nerve recordings. Analysis of mutual information between pairs of fascicles shows that 1.4 bits of information are shared among these fascicles, meaning we can determine one fascicle’s state (activity) based on another to some extent. Thus, not all recorded information is independent between fascicles, with only 2.6/4 bits of unique (independent) information being recorded by CNTY electrodes from each fascicle.

CNTY electrodes outperform current state-of-the-art electrodes in recording quality, stability, and selectivity. However, like carbon fiber electrodes [41], the number of contacts is limited to one per electrode. Some state-of-the-art electrodes, such as TIME and tf-LIFE, have multiple contact points per electrode, resulting in higher yield. Therefore, a multiple-channel recording system with CNTY electrodes would require multiple implants. While the CNTY electrode only has one contact per electrode, implanting these electrodes in each fascicle of a human nerve would yield ∼20–30 individual channels, providing a total of 52–78 bits (20*2.6 bits) of independent information encoded in spiking activity. Current commercially available myoelectric control prosthetics use either one or two channels for control, where one channel encodes for two states on/off, equivalent to one bit [42]. Thus, having 52–78 bits could in theory control an upper limb prosthesis with 27 degrees of freedom with each degree of freedom having three states that need encoding. Increasing the number of states that need to be encoded for each degree of freedom for finer joint control would increase the number of bits needed to encode each state, requiring more information from neural recordings. Ongoing CNTY electrode design improvements will be crucial for implanting these electrodes in large animal models where the number of fascicles per nerve is significantly higher than that of a rat and similar to human nerves.

Overall, CNTY electrodes demonstrated the ability to record neural signals with a high SNR of 15 dB, significantly greater than the 6 dB calculated using reported values for tf-LIFE electrodes. These electrodes showed no significant decrease in performance over 12 weeks. Furthermore, 12 bits of information could be extracted from the whole nerve, significantly greater than the 3 bits extractable using the FINE electrode. Of the 12 bits, 7.8 bits were independent and 4.2 bits were shared between the three fascicles. This technology could be used to develop PNIs that interface with many fascicles of human nerves and provide reliable long-term neural recordings with sufficient information content to drive neural prosthesis control.

## Data Availability

The data recorded and the code used for analysis in this study are available upon reasonable request from the corresponding author.
